# Tomato Domestication Attenuated Responsiveness to a Beneficial Soil Microbe for Plant Growth Promotion and Induction of Systemic Resistance to Foliar Pathogens

**DOI:** 10.3389/fmicb.2020.604566

**Published:** 2020-12-18

**Authors:** Amit K. Jaiswal, Tesfaye D. Mengiste, James R. Myers, Daniel S. Egel, Lori A. Hoagland

**Affiliations:** ^1^Department of Horticulture and Landscape Architecture, Purdue University, West Lafayette, IN, United States; ^2^Department of Botany and Plant Pathology, Purdue University, West Lafayette, IN, United States; ^3^Department of Horticulture, Oregon State University, Corvallis, OR, United States

**Keywords:** induce systemic resistance, plant growth, tomato domestication, beneficial microbes, control efficacy, gray mold, late blight, phenolic contents

## Abstract

Crop domestication events followed by targeted breeding practices have been pivotal for improvement of desirable traits and to adapt cultivars to local environments. Domestication also resulted in a strong reduction in genetic diversity among modern cultivars compared to their wild relatives, though the effect this could have on tripartite relationships between plants, belowground beneficial microbes and aboveground pathogens remains undetermined. We quantified plant growth performance, basal resistance and induced systemic resistance (ISR) by *Trichoderma harzianum*, a beneficial soil microbe against *Botrytis cinerea*, a necrotrophic fungus and *Phytophthora infestans*, a hemi-biotrophic oomycete, in 25 diverse tomato genotypes. Wild tomato related species, tomato landraces and modern commercial cultivars that were conventionally or organically bred, together, representing a domestication gradient were evaluated. Relationships between basal and ISR, plant physiological status and phenolic compounds were quantified to identify potential mechanisms. *Trichoderma* enhanced shoot and root biomass and ISR to both pathogens in a genotype specific manner. Moreover, improvements in plant performance in response to *Trichoderma* gradually decreased along the domestication gradient. Wild relatives and landraces were more responsive to *Trichoderma*, resulting in greater suppression of foliar pathogens than modern cultivars. Photosynthetic rate and stomatal conductance of some tomato genotypes were improved by *Trichoderma* treatment whereas leaf nitrogen status of the majority of tomato genotypes were not altered. There was a negative relationship between basal resistance and induced resistance for both diseases, and a positive correlation between *Trichoderma*-ISR to *B. cinerea* and enhanced total flavonoid contents. These findings suggest that domestication and breeding practices have altered plant responsiveness to beneficial soil microbes. Further studies are needed to decipher the molecular mechanisms underlying the differential promotion of plant growth and resistance among genotypes, and identify molecular markers to integrate selection for responsiveness into future breeding programs.

## Introduction

Tomato (*Solanum lycopersicum L.*) is currently the second most important vegetable crop grown in the world and its production is growing rapidly, with the total area under cultivation having doubled during the last two decades^1^. This important vegetable crop is also widely used as a model for investigating plant development and pest resistance mechanisms because of its relatively short reproductive cycle, availability of a fully sequenced genome, and a variety of mutants which allow investigation of individual plant traits. Tomato (*Solanum* spp.) originated from western South America along the Andes in Peru, Ecuador and Chile, and the Galapagos Islands (Bergougnoux, 2014). Modern tomato is thought to have been domesticated from its wild ancestors (*S. pimpinellifolium* and *S. lycopersicum* var. *cerasiforme*), and then selected for adaptive traits in local agronomic environments (Zhu et al., 2018). The exact site of tomato domestication is unclear, but it is thought to have occurred in Mexico or Peru (Bai and Lindhout, 2007; Bergougnoux, 2014). Before the Spanish transported tomato from Mexico to Europe in the 15th century, its domestication was in a fairly advanced phase, although over the past centuries, further selection occurred at a much more intense level throughout Europe and the rest of the world (Sims, 1979). The combination of early domestication events followed by targeted breeding practices have resulted in the development of modern tomato cultivars with diverse agronomic traits including improved fruit characteristics (set, size, shape, color, firmness, shelf-life, phenolic contents) and plant growth habits (self-pruning, height, and earliness) (Bai and Lindhout, 2007; Causse et al., 2007; Bauchet and Causse, 2012). However, these practices have also resulted in a strong reduction in the genetic diversity of modern cultivars compared to their wild relatives. For instance, basal defenses against pests have been weakened because of selection for higher yield and fruit quality may have come at the expense of defense traits (Chen et al., 2015).

Tomato plants are susceptible to over 200 pests and diseases caused by pathogenic fungi, bacteria, viruses and nematodes (Lukyanenko, 1991). Over evolutionary time, plants have developed constitutive and induced defense mechanisms to defend against these pathogens (War et al., 2012; Boots and Best, 2018). Constitutive (preformed) defenses include mechanical barriers (such as cell walls, waxy epidermal cuticles, stomatal structure, trichomes, and bark), and biochemical barriers (primary and secondary metabolites) that are always present in the plant. In contrast, induced defense mechanisms, or induced resistance (IR) responses, are activated in response to pathogen attack, and thus are less energetically expensive. IR is initiated by a specific stimulus, which allows plants to limit subsequent pest challenges (Vallad and Goodman, 2004). Among IR responses that are activated against a broad range of pests and pathogens are systemic acquired resistance (SAR), and induced systemic resistance (ISR). SAR, which is generally initiated by a hypersensitive reaction resulting from a local infection, is thought to be mediated by salicylic acid and involve the synthesis of PR (pathogenesis-related) proteins. SAR can be triggered by both biological and chemical elicitors [such as 2,6-dichloroisoniciotinic acid (INA), acibenzolar-*S*-methyl (BTH), and β-aminobutyric acid (BABA)] (Vallad and Goodman, 2004; Vlot et al., 2009). In contrast, ISR is generally activated by plant growth-promoting rhizobacteria (PGPR) and fungi (PGPF) and is thought to be mediated by the phytohormones jasmonic acid and ethylene (Vallad and Goodman, 2004; Pieterse et al., 2014). Consequently, the plant microbiome is now widely regarded as a key determinant of plant health, and interest in identifying strategies that can enrich beneficial microbial communities to increase plant productivity and suppress diseases is growing rapidly. *Trichoderma* spp. are one group of microbes that have received much attention for their potential to stimulate plant growth and ISR. *Trichoderma* spp., which are members of the Ascomycetes fungi and opportunistic symbionts, have been widely used as biopesticides and biofertilizers in agricultural fields. Currently, more than 60% of all registered biopesticides contain a single *Trichoderma* isolate or mixture of *Trichoderma* species (Lopez-Bucio et al., 2015). The positive influence of *Trichoderma* on host plants could be related to several mechanisms, including ISR, but this is still not well understood.

Previous studies investigating direct plant defense responses have demonstrated that domesticated genotypes are often more susceptible to diseases (Mieslerova et al., 2000; Foolad et al., 2014; Smith et al., 2014) and insect pests (Chaudhary, 2013; Chen et al., 2015) than their wild relatives, due to the presence of individual resistance genes (Mieslerova et al., 2000; Foolad et al., 2014; Smith et al., 2014). Consequently, plant breeders commonly identify genes from wild crop relatives and introgress them into modern germplasm to enhance disease resistance. A few studies have observed genotype-specific responses to biological and chemical inducers that stimulate plant growth and IR against pests in tomato (Ishikawa et al., 2007; Tucci et al., 2011; Sharma et al., 2012), indicating that these relationships are also under genetic control and could potentially be exploited in breeding programs. However, how tomato domestication and targeted breeding efforts have influenced tripartite relationships between aboveground plant pathogens and belowground beneficial soil microbes that mediate systemic resistance (indirect responses), remains undetermined. We hypothesized that responsiveness to beneficial microbes would be reduced during domestication because it represents a physiological cost to the plant, and explicit selection/development of resistant cultivars and the farming conditions under which new tomato cultivars are developed would also affect responsiveness to microbes that trigger ISR. For example, selection for high yield in farming systems that rely on synthetic fertilizers and pesticides could have decoupled relationships between beneficial microbes in the rhizosphere from plant fitness traits since these relationships can come at a cost to the plant in terms of carbon allocation and the need to temporally relax defense processes (Hoagland, 2015). If this has occurred, then plants would not be as responsive to biological inducers that can enhance plant growth and protect plants from a broad range of pathogens and insect pests. To test this hypothesis, we quantified changes in plant growth and responsiveness to ISR by *T. harzianum* in diverse tomato genotypes, against *Botrytis cinerea* and *Phytophthora infestans* that have distinct infection strategies. *B. cinerea* is a necrotrophic Ascomycetes fungus that causes the gray mold disease whereas, *Phytophthora infestans* is a hemi-biotrophic Oomycete that cause late blight disease. *B. cinerea* and *P. infestans* are among the most destructive pathogens of tomatoes under greenhouse and field production respectively, causing up to 50% to entire crop losses under ideal environmental conditions (Nowicki et al., 2012; Elad et al., 2016). Total phenolic and flavonoid contents were quantified to determine relationships between these compounds and variation in disease severity, in order to ascertain if this could be a mechanism responsible for ISR. Flavonoids and phenolics are important secondary metabolites and bioactive compounds in plants, and several phenolic compounds affect plant defenses against diseases by directly inhibiting the pathogen or by reinforcing plant cell walls acting as a mechanical barrier (Candela et al., 1995; de Ascensao and Dubery, 2003).

## Materials and Methods

### Tomato Plants and Growth Conditions

Twenty-five diverse tomato genotypes were included in this study to represent a gradient of domestication in tomato (Table 1). This included several wild relatives of tomato including *S. pimpinellifolium*, which is thought to be represent the original source of modern-day tomato. These accessions are not expected to have undergone any selection by humans. Several accessions of *S. lycopersicum* var. *cerasiforme* were included to represent an intermediate step in tomato domestication, where the wild ancestor was first selected for larger fruit and more fruit per plant. Finally, a set of landraces, heirlooms, and modern commercial cultivars (conventionally or organically bred) were included to represent a continuum of the final stages of domestication, where tomatoes were further selected for specific agronomic properties, and have been increasingly conducted using more rigorous processes such as crossing with wild ancestors to introgress specific genes associated with disease resistance. All genotypes were screened for changes in plant growth and systemic resistance induced by *T. harzianum* against *B. cinerea* and *P. infestans*. We define heirlooms as open-pollinated cultivars that have been passed down for several generations; and landraces as domesticated, locally adapted and genetically heterogeneous cultivars that were improved by early agricultural methods. Seed of wild accessions and landraces were obtained from the C. M. Rick Tomato Genetics Resource Center (TGRC), University of California, Davis (LA numbers), and R. G. Gardner, North Carolina State University, Mills River, NC (PI numbers). Remaining seeds were obtained from commercial seed companies, B. Horneberg, Georg-August-Universitat Gottingen, Germany, or our on-going organic breeding program^2^. Tomato seeds were surface sterilized with 2.7% NaOCl for 30 min and rinsed three times with sterile water. Seeds were sown in a tray containing pasteurized potting media, peat: perlite (80:20 v:v; Midwest Trading, IL, United States; pasteurization at 70°C for 24 h), and placed under a mist irrigation system. After 2 weeks, a single tomato seedling was transplanted to each pot (0.5 L) containing the pasteurized potting media, *T. harzianum* was applied as a soil drench (details below) to seedling roots, and tomato plants were grown in a glasshouse for another 3 weeks at 24 ± 1°C and fertilized and irrigated with drippers (Arrow Dripper 2.3 lt/h, Netafim, Israel) four times per day for 10 s, allowing for 25–50% drainage. Each treatment included six biological replicates.

**TABLE 1:** Tomato genotypes used in present study.

Botanical name	Categories	TGRC no./cultivar	Origin	Seeds source	Growth habit
*S. lycopersicoides*	Wild relatives	LA2951	Chile	TGRC	
*S. pennellii*	Wild relatives	LA0716	Peru	TGRC	
		LA1926	Peru	TGRC	
*S. chilense*	Wild relatives	LA1932	Peru	TGRC	
*S. habrochaites*	Wild relatives	LA1223	Ecuador	TGRC	Indeterminate
*S. pimpinellifolium*	Wild relatives	LA1589	Peru	TGRC	Indeterminate
		PI224710	Mexico	From R. Gardener	Indeterminate
*S. lycopersicum var. cerasiforme*	Wild tomato	LA1231	Ecuador	TGRC	Indeterminate
	Cherry tomato	LA1268	Columbia	TGRC	Indeterminate
		LA2845	Peru	TGRC	Indeterminate
		Matt’s wild cherry	Mexico	From R. Gardener	Indeterminate
*S. lycopersicum*	Landrace	LA0134C	Peru	TGRC	Indeterminate
	Heirloom	Brandywine	United States	Johnny’s selected seeds company	Indeterminate
		Corbarino	Europe (Italy)	Reimer seeds company	Indeterminate
	Early Modern	Crimson Sprinter	United States	High mowing seeds company	Semi-determinate
		Wisconsin 55	United States	Johnny’s selected seeds company	Indeterminate
		M-82		TGRC	Determinate
	Hybrids (F1)	Iron Lady	United States	High mowing seeds company	Determinate
	Inbred	NC2CELBR	United States	R. Gardener	Determinate
	Organic OP	Primavera	Europe (Germany)	B. Horneburg	Indeterminate
		Clou	Europe (Germany)	B. Horneburg	Indeterminate
		T-1807^#*a*^	United States	TOMI	Indeterminate
		T-1809^#*b*^	United States	TOMI	Indeterminate
		T-1815^#*c*^	United States	TOMI	Indeterminate
		T-1820^#*d*^	United States	TOMI	Indeterminate

*^#^Pedigree information of tomato genotypes from our on-going organic breeding program (https://eorganic.info/tomi). ^*a*^(((((CSxNC2)@2)x(((W55xNC1)xW55)@2))@ C-9) NCSU #1)@@. ^*b*^((((((W55xNC1)xW55)@2)x((CSxNC2)@2))@ F-1) IN #1)@@. ^*c*^(((((W55xNC1)xW55)@3)@ G-1) IN #4)@@. ^*d*^(((((W55xNC1)@2)x(((W55xNC1)xW55)@2))@ A-10) WI #6)@@.*

### Treatment With *Trichoderma harzianum*

*Trichoderma harzianum* (T-22) was isolated from a commercial product (RootShield-Bioworks, Inc., Geneva, NY, United States) and the identity of the isolate was confirmed by PCR and Sanger sequencing using ITS1F and ITS4 primer sets (Bellemain et al., 2010). *T. harzianum* was cultured on potato dextrose agar in petri dishes at 24°C for 8 days and then conidia were harvested with sterile distilled water and filtered through four-layers of cheesecloth. The concentration of the conidia was determined using a hemocytometer under a light microscope and the concentration was adjusted to 1 × 10^7^ mL^–1^. Conidial suspensions (10 mL) were applied as a soil drench near the root zone immediately following tomato transplantation, and again 4 h before tomato leaves were excised for detached leaf disease bioassays.

### Pathogens Inoculum and Disease Bioassays

*Botrytis cinerea* (B05.10) was grown on 2 × V8 agar medium (360 mL V8 juice, 2 g CaCO_3_ and 20 g Bacto-agar in 1 L medium) at 24°C for 14 days at 12/12 light/dark cycle (Smith et al., 2014). Inoculum of *B. cinerea* was prepared by cutting blocks of agar media and agitating them in 1% Sabouraud maltose broth (SMB). Conidia were separated from the agar and mycelium by filtering through sterile cheesecloth and spore concentrations were adjusted to 5 × 10^4^ mL^–1^ with 1% SMB.

*P. infestans* (US-23, kindly provided by Prof. William E. Fry, Cornell University, Ithaca, NY, United States) was grown on Rye-B agar medium (in 1 L medium 15 g Bacto-agar, 20 g sucrose 0.05 g β-sitosterol, and supernatant from 60 g of boiled rye grain that were soaked overnight in water at 18°C for 20 days in dark) (Foster et al., 2009). *P. infestans* inoculum was obtained by gently washing the Rye-B agar plates with 3 mL 0.1% water agar. The sporangial suspension of *Phytophthora* was placed in a refrigerator for 2 h to induce zoospore release and then filtered through sterile cheesecloth and adjusted to 1 × 10^4^ mL^–1^ spore concentration.

To evaluate basal and ISR mediated by *Trichoderma*, the third and fourth leaves of the tomato plants were detached and inoculated with *B. cinerea* and *P. infestans* in separate assays. Three to four drops of *B. cinerea* (5 μL each) and *P. infestans* (20 μL each) spore suspensions were used to inoculate tomato leaves, which were placed on sterile filter paper moistened with sterile water and kept in a covered transparent plastic box. Inoculated leaves were incubated in a growth chamber with a 14/10 light/dark cycle at 24°C and 18°C for *B. cinerea* and *P. infestans*, respectively. Disease severity was quantified daily by taking pictures of infected areas using a digital camera and measuring the infected areas using ImageJ software^3^. Final disease values in *Trichoderma* treated (T) and untreated control (C) plants were used to calculate disease control efficacy (CE,%) by the treatments:

$$CE,\%=\left(\frac{C-T}{C}\right)\times 100$$

### Tomato Growth, Nutrient Concentration, and Physiology Parameters

Tomato growth parameters were evaluated at the end of the experiments (28 DAT). Plant height was measured from the base of the stem to the top of the uppermost leaflet. Total number of leaves and fresh weight of roots were also measured at harvest. Plant shoots were washed with distilled water and then dried in an air circulating (60°C) oven for 7–10 days until dry weight was unchanged to quantify dry shoot weight. Dried shoot tissue was ground to a fine powder, and approximately 30 mg of tissue was analyzed for carbon (C) and nitrogen (N) by combustion (CHN 2000; LECO Corp., St. Joseph, MI, United States).

Net photosynthesis rate (An), stomatal conductance (Gs), transpiration rate (Tr), and electron transport rate (ETR) were measured in 15 randomly selected tomato genotypes from 9:00 to 11:00 h using a portable photosynthesis system (Li-6400XT, LI-COR Inc., Lincoln, NE, United States) at leaf temperature 30°C, light 1200 μmol m^–^2 s^–1^, 10% blue light, 400 μmol CO2 mol^–1^ (Jaiswal et al., 2017; Paudel et al., 2017).

### Total Phenolic and Flavonoid Contents

Total phenolic content (TPC) and total flavonoid content (TFC) were analyzed as previously described (Ainsworth and Gillespie, 2007; Sulaiman and Balachandran, 2012) with a few modifications. Briefly, a frozen tomato leaf was ground to a fine powder with a tissue homogenizer (SPEX SamplePrep 1600 MiniG Metuchen, NJ, United States) and 25 mg of the powdered leaf was homogenized in 1.6 mL of 80% methanol and vortexed for 30 min. The suspension was centrifuged for 5 min at 13,000 × *g* at room temperature and the supernatant (1000 μL) was collected.

To quantify the TPC, an aliquot of 100 μL of each sample, a standard (0–300 μg mL^–1^ gallic acid in 80% methanol), or blank (methanol), were reacted with 200 μL Folin-Ciocalteu (10%) reagent and 800 μL 700 mM Na_2_CO_3_ for 2 h in the dark at room temperature. The reaction products (200 μL) were measured at λ = 765 nm using a microplate reader spectrophotometer (Epoch Microplate Spectrophotometer, BioTek Instruments, Inc., VT, United States) and the total phenolic concentration was expressed as mg gallic acid g^–1^FW.

To quantify the TFCs, an aliquot of 100 μL of each sample, a standard (0–100 μg mL^–1^ quercetin in 80% methanol), or a blank (methanol) were mixed with 400 μL distilled water and then 30 μL of 5% NaNO_2_ was added. After 5 min, 30 μL of 10% AlCl_3_ was added and mixed. After 5 min, 200 μL 1 M NaOH was added and the volume was increased to 1 mL with distilled water (240 μL). The mixtures were allowed to stand for 15 min. The reaction product (200 μL) was measured at λ = 510 nm using a microplate reader spectrophotometer (Epoch Microplate Spectrophotometer, BioTek Instruments, Inc., VT, United States) and TFC was calculated as quercetin equivalents g^–1^FW.

### Experimental Design and Statistical analysis

All plant experiments were conducted twice, and the two experimental replicates were pooled and analyzed jointly with ‘experiment’ as an additional factor. Data were analyzed by ANOVA (analysis of variance) using JMP Pro 14 software (SAS Institute, Cary, NC, United States). To quantify the domestication gradient effect, the tomato genotypes were grouped into the following categories: (1) wild relatives (*S. lycopersicoides*, *S. pennellii*, *S. chilense*, *S. habrochaites*), (2) *Pimpinellifolium* wild tomato (*S. pimpinellifolium*)- first domestication, (3) *Cerasiforme* wild tomato (*S. lycopersicum* var. *cerasiforme*)- second domestication; intermediate between *S. pimpinellifolium and S. lycopersicum*, (4) Landrace/Heirloom (*S. lycopersicum)*, and (5) modern tomato (*S. lycopersicum*: early modern open-pollinated varieties, hybrids (F1), inbreds, and organic open-pollinated varieties). Similarly, to analyze the effect of breeding system, the modern tomato genotypes were grouped into the following categories (1) conventionally bred tomato [early modern open-pollinated varieties, hybrids (F1), inbreds], and (2) organically bred tomato (organic open-pollinated varieties). Multiple comparisons of the means were conducted using the Tukey-Kramer (HSD) test (α = 0.05).

## Results

### Effect of Domestication and Breeding on Basal Plant Growth

**FIGURE 1:**
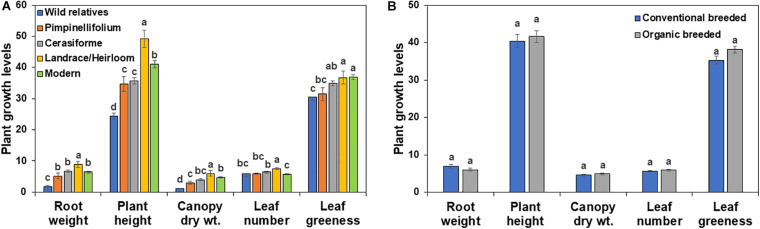
Effects of tomato **(A)** domestication and **(B)** breeding conditions on basal plant growth and development in diverse tomato groups (see Table 1). For each tomato genotypes six biological replicates were included per treatment. Root weight, plant height, canopy dry weight, leaf number and leaf greenness are presented in g, cm, g, count and Spad meter value, respectively. Columns labeled by a different letter are significantly different at *P* ≤ 0.05 according to Tukey-Kramer HSD test within each individual growth parameter.

**FIGURE 2:**
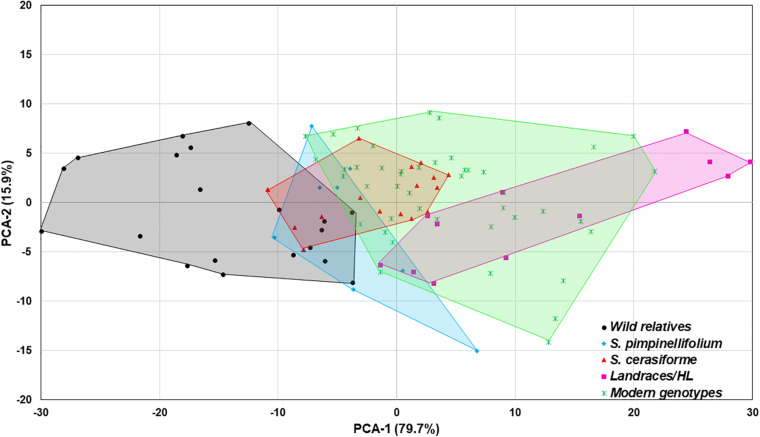
Principal component analysis (PCA) of all plant phenotype parameters. The percentage of variance explained by each component is displayed in parentheses. The wild relatives, *Pimpinellifolium*, *S. cerasiforme* wild tomato, landraces/heirloom and modern genotypes are depicted in black, light blue, green, dark blue, and orange, respectively.

**FIGURE 3:**
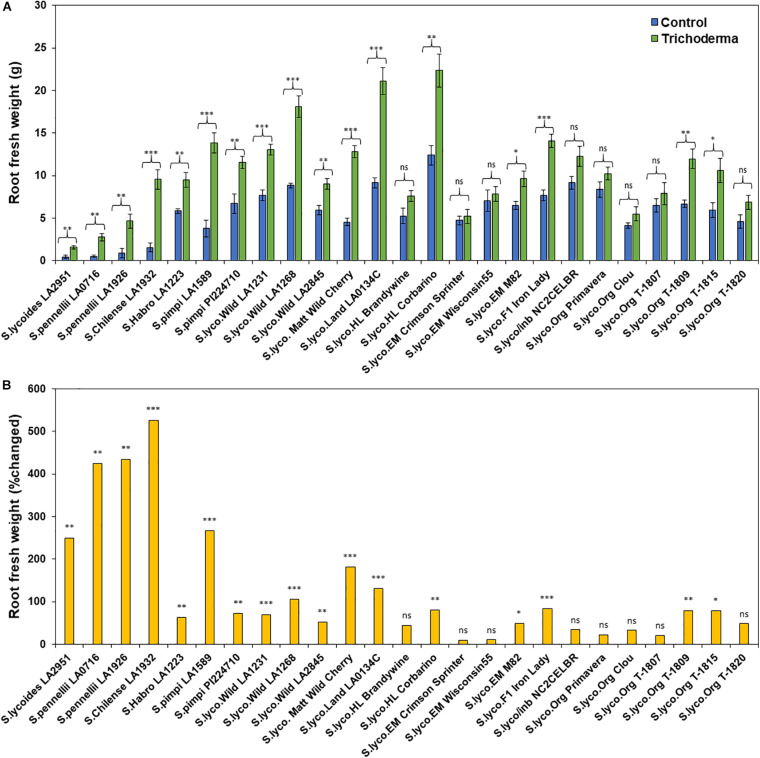
Effect of *Trichoderma* treatment on **(A)** root biomass (g) in a diverse set of tomato genotypes. Graph **(B)** represents the percentage in root biomass changed by *Trichoderma* treatment in comparison with the control treatment. Asterisks indicate significant differences in root biomass in the *Trichoderma*-treated versus control plants using the Student’s *t*-test at a ^∗^*P* ≤ 0.05, ^∗∗^*P* ≤ 0.01 and ^∗∗∗^*P* ≤ 0.001. Bars represent the standard error.

**FIGURE 4:**
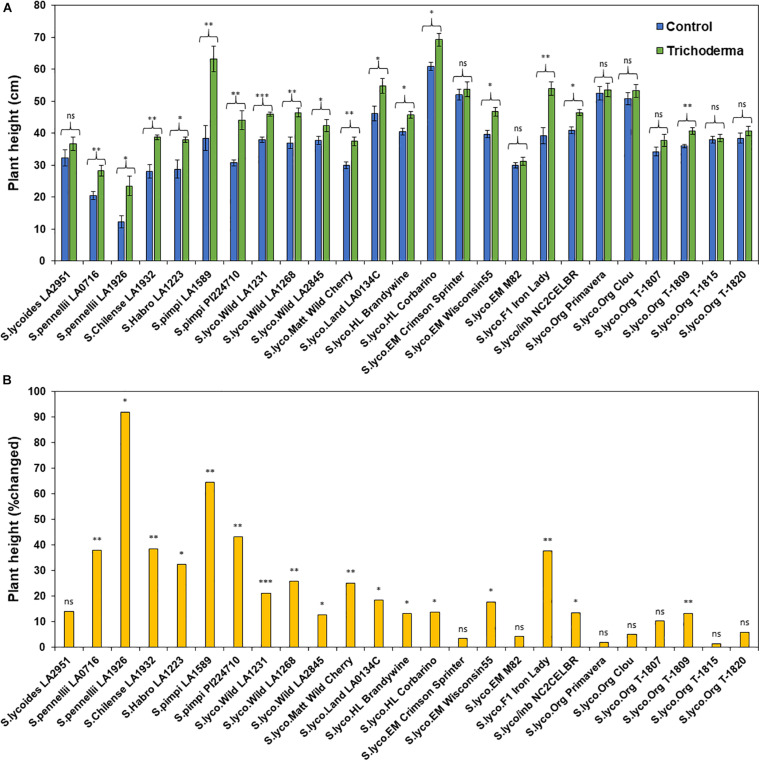
Effects of *Trichoderma* treatment on **(A)** plant height (cm) in a set of diverse tomato genotypes. Graph **(B)** represents the percentage in plant height changed by *Trichoderma* treatment in comparison with the control treatment. Asterisks indicate significant differences in plant height in the *Trichoderma*-treated versus control plants using the Student’s *t*-test at a ^∗^*P* ≤ 0.05, ^∗∗^*P* ≤ 0.01 and ^∗∗∗^*P* ≤ 0.001. Bars represent the standard error.

In the absence of *Trichoderma*, the one-way ANOVA model indicated that tomato domestication had a significant effect on all above- and below-ground plant growth parameters (root weight, plant height, canopy dry weight, number of leaves, and leaf greenness) evaluated (*P* < 0.0001). In general, landrace/heirloom and modern tomatoes had greater early plant growth compared to wild relatives and progenitors of cultivated tomato (Figures 1A, 3A, 4A). Organically bred genotypes had slightly greater growth compared to the conventionally bred genotypes, though the effects of breeding conditions (conventional vs. organic) were not statistically significant (*P* = 0.0756, Figure 1B). Principal component analyses (PCA) of all the growth parameters indicated a clear separation between wild relatives, *Pimpinellifolium* and modern tomato genotypes, representing 79.7 and 15.9% of the variance explained by the first and second principal component (PC1 and PC2), respectively (Figure 2). There was some overlap between *S. cerasiforme*, landrace/heirloom and modern tomato genotypes.

### Effect of Domestication and Breeding on *Trichoderma*-Induced Plant Growth

The application of *Trichoderma* significantly increased root biomass in 17 out of 25 genotypes (Figure 3A and Supplementary Table 3). In responsive genotypes, root biomass was increased by up to 526% in comparison with non-treated control plants. Similarly, plant height was also significantly greater in 17 genotypes by up to 91.8%, in comparison with non-treated control plants (Figures 4A,B and Supplementary Table 3). The other eight genotypes were non-responsive to the presence of *Trichoderma*, and none of the genotypes were negatively affected by *Trichoderma* treatment. Canopy dry weight and number of leaves per plant had a similar pattern (Data not shown). In both parameters (root biomass and plant height) the wild relatives, *S. cerasiforme*, landraces and heirlooms were most responsive, while modern genotypes (conventional and organically bred lines) were less responsive or non-responsive to the *Trichoderma* treatment. Overall, the responsiveness of tomato plants toward increased plant growth in response to the *Trichoderma* treatment gradually decreased along the domestication gradient, and organically bred genotypes were slightly greater than other modern germplasm (Figures 3B, 4B). Leaf greenness estimated using a SPAD meter (proxy of chlorophyll content), was significantly greater in 15 *Trichoderma*-treated tomato genotypes by up to 25% than their respective non-treated control plants (Supplementary Figures 1A,B).

One-way ANOVA analyses of the percentage change in plant growth parameters by *Trichoderma* also indicated that the domestication gradient had a significant effect on *Trichoderma*-induced plant growth parameters (*P* < 0.0001), with the exception of leaf greenness (*P* = 0.2953). Overall, responsiveness was greater in tomato from wild relatives in general, followed by *Pimpinellifolium* > *S. cerasiforme* > landrace/heirloom > modern tomato. The breeding conditions did not have any significant effect (percentage changed) of *Trichoderma* treatment for all tested plant growth parameters. Furthermore, the two-way ANOVA model applied to investigate interactions between *Trichoderma* treatment and tomato domestication were all highly significant (Supplementary Table 1). The factors “*Trichoderma* treatment” and “domestication gradient” had significant effects on all tested growth parameters. However, the interaction between these two main factors was only significant for root biomass and plant height (Supplementary Table 1). Similarly, two-way ANOVA analyses indicated that the interaction between breeding categories and *Trichoderma* treatment on growth parameters was not significant (Supplementary Table 2).

### Effect of Domestication and Breeding on Basal Resistance

**FIGURE 5:**
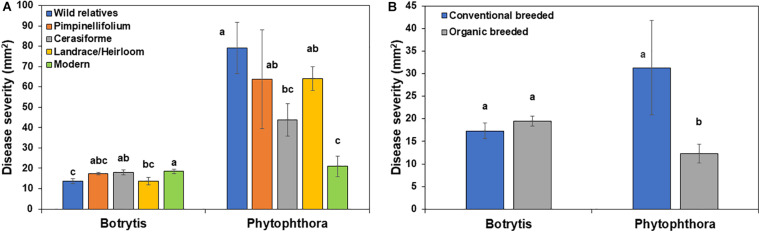
Effect of tomato **(A)** domestication and **(B)** breeding conditions on basal resistance against *Botrytis cinerea* and *Phytophthora infestans*. Columns labeled by a different letter are significantly different at *P* ≤ 0.05 according to Tukey-Kramer HSD test within a disease.

In the absence of *Trichoderma*, the one-way ANOVA model indicated that domestication and breeding category had a significant effect on tomato basal defense against gray mold (*P* = 0.0182) and late blight (*P* < 0.0001) disease severity. Wild relatives of tomato were more resistant to *Botrytis* compared to other groups, while, wild relatives, *Pimpinellifolium*, *S. cerasiforme* and landrace/heirloom were more susceptible to *P. infestans* compared to modern cultivars (Figure 5A). There was no significant difference in gray mold disease severity between conventional and organically bred tomato genotypes (*P* = 0.3066; Figure 5B), however, organically bred tomato genotypes were more resistant to late blight compared to the conventionally bred genotypes (*P* < 0.05; Figure 5B).

**FIGURE 6:**
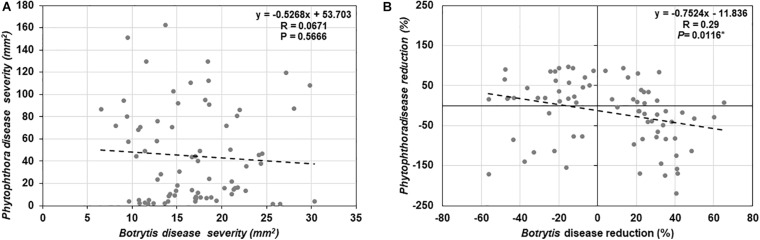
Correlation analysis between **(A)** basal resistance against *Botrytis cinerea* and *Phytophthora infestans* disease, **(B)** induced resistance against *B. cinerea* and *P. infestans* by *Trichoderma* treatment in comparison with the control treatment in 25 diverse tomato genotypes.

To determine if trade-offs between basal defense against a necrotrophic (*B. cinerea*) and a hemi-biotrophic (*P. infestans*) fungal pathogen exists among the 25 tomato genotypes evaluated in this study, a correlation analysis was conducted (Figure 6A), however, there was no significant relationship detected (*R* = −0.0671, *P* = 0.5666).

### Effect of Domestication and Breeding on *Trichoderma*-Induced Systemic Resistance

**FIGURE 7:**
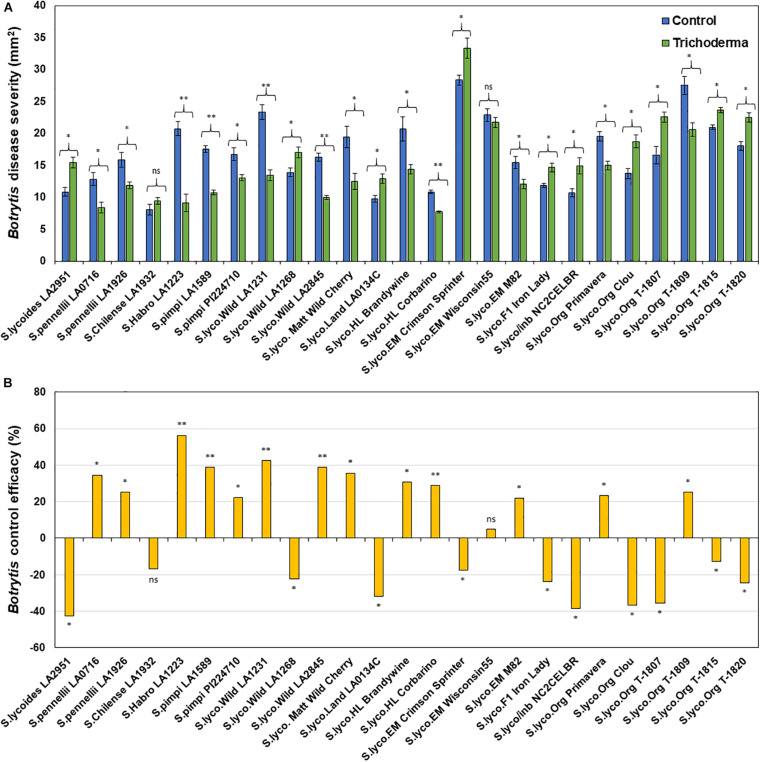
Effect of *Trichoderma* treatment on plant resistance against *Botrytis cinerea* in a diverse set of tomato genotypes **(A)**. Graph **(B)** represents control efficacy of *Trichoderma* against *B. cinerea* in comparison with the control treatment. Asterisks indicate significant differences in disease severity in the *Trichoderma*-treated versus control plants using the Student’s *t*-test at a ^∗^*P* ≤ 0.05 and ^∗∗^*P* ≤ 0.01. Bars represent the standard error.

**FIGURE 8:**
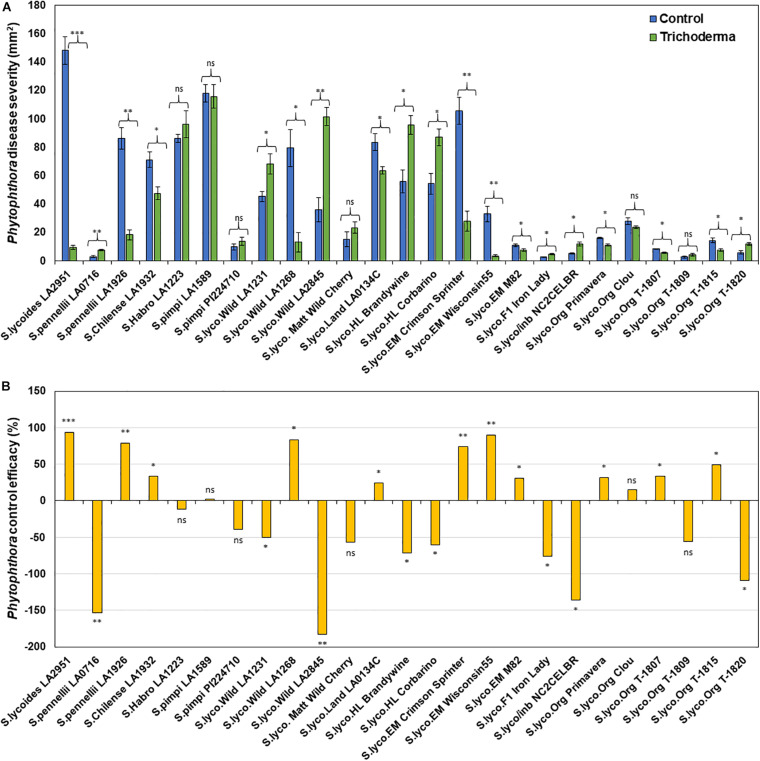
Effect of *Trichoderma* treatment on plant resistance against *Phytophthora infestans* in a diverse set of tomato genotypes **(A)**. Graph **(B)** represents control efficacy of *Trichoderma* against *P. infestans* in comparison with the control treatment. Asterisks indicate significant differences in disease severity in the *Trichoderma*-treated versus control plants using the Student’s *t*-test at a ^∗^*P* ≤ 0.05, ^∗∗^*P* ≤ 0.01 and ^∗∗∗^*P* ≤ 0.001. Bars represent the standard error.

Application of *Trichoderma* significantly reduced gray mold disease severity in 13 out of the 25 genotypes (*P* < 0.05; Figures 7A,B and Supplementary Table 4). In plants that responded to the *Trichoderma* treatment, gray mold was reduced by up to 56% relative to non-treated control plants. In two genotypes, there was no effect of *Trichoderma* treatment, and gray mold disease severity was significantly greater in 10 genotypes by up to 43% in comparison with the respective non-treated control plants (*P* < 0.05; Figures 7A,B). Similarly, late blight disease severity was also significantly reduced in 11 tomato genotypes by up to 94% in comparison with the respective non-treated control plants (*P* < 0.05; Figures 8A,B and Supplementary Table 4). Six were non-responsive to *Trichoderma* treatment, and in eight genotypes, disease severity by late blight was significantly increased by up to 183% (*P* < 0.05; Figures 8A,B). Overall, induction of defense responses by *Trichoderma* tended to be stronger against *B. cinerea* (+94 to −183%) than for *P. infestans* (+56 to −43%).

Results of the one-way ANOVA analysis comparing the efficacy of *Trichoderma* induced control against *B. cinerea* and *P. infestans* indicated that the domestication gradient had a significant effect on *Trichoderma*-ISR to *B. cinerea* (*P* = 0.0026) but not *P. infestans* (*P* = 0.3110). Overall, ISR to *B. cinerea* was greatest in *S. cerasiforme* followed by *Pimpinellifolium* > wild relatives > landrace/heirloom > modern tomato genotypes but there was no significant difference between breeding categories on *Trichoderma-*ISR to either disease (*P* > 0.05). All two-way ANOVA models applied to investigate interactions between *Trichoderma* treatment and tomato domestication on the two diseases were highly significant (Supplementary Table 1). The factors “*Trichoderma* treatment” and “domestication gradient” had significant effects on both diseases. Moreover, the interaction between these two main factors was also significant for both diseases (Supplementary Table 1). However, the interaction between breeding categories and *Trichoderma* treatment on diseases caused by both pathogens had non-significant effects (Supplementary Table 2). To determine if there was a trade-off between *Trichoderma*-ISR to a necrotrophic fungus (*B. cinerea*) and a hemi-biotrophic oomycete (*P. infestans*), a correlation between control efficacy (disease reduction) was conducted among the 25 tomato genotypes evaluated (Figure 6B). Interestingly, we observed a significant negative correlation (weak correlation) between *Botrytis* and *Phytophthora* disease reduction by *Trichoderma* (*R* = −0.29, *P* = 0.0116).

**FIGURE 9:**
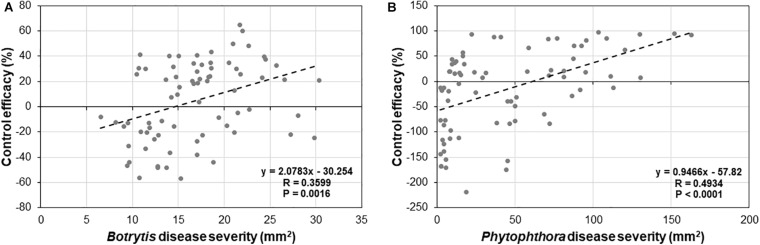
Correlation analysis between basal and induced resistance by *Trichoderma* treatment against **(A)** *B. cinerea*, and **(B)** *P. infestans* in 25 diverse tomato genotypes.

To determine if there is any relationship between levels of basal and induced resistance by *Trichoderma*, a correlation analysis between basal disease and control efficacy of *Trichoderma* for *B. cinerea* (Figure 9A) and *P. infestans* (Figure 9B) was conducted among the 25 tomato genotypes. We found a strong negative correlation between basal resistance and induced resistance for both *Botrytis* (*R* = 0.3599, *P* = 0.0016) and *P. infestans* (*R* = 0.4934, *P* < 0.0001).

### Effects of *Trichoderma* Treatment on Tomato Physiological Parameters and Nutrient Content

**FIGURE 10:**
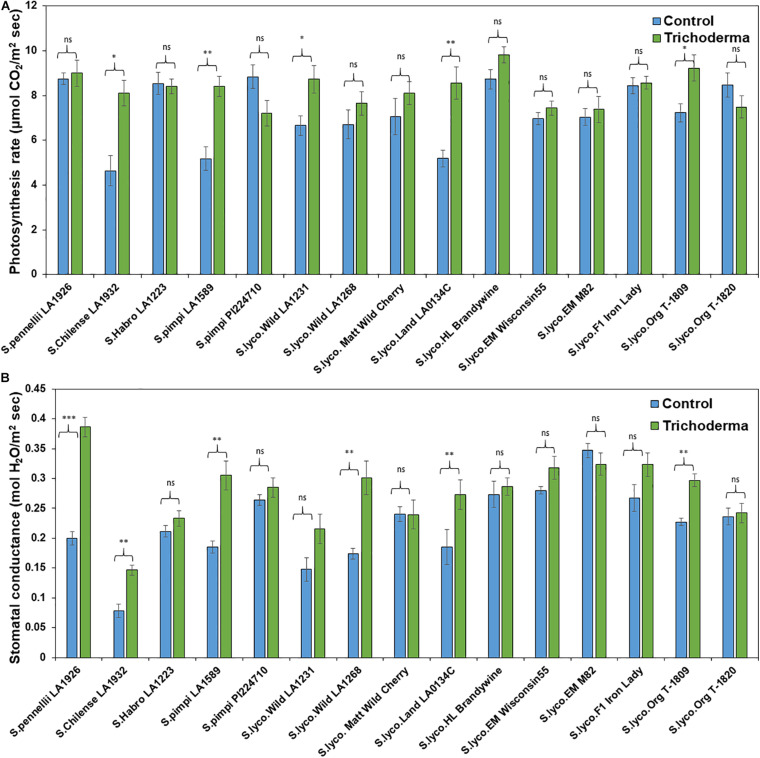
Effects of *Trichoderma* treatment on **(A)** photosynthesis and **(B)** stomatal conductance in a set of diverse tomato genotypes. Asterisks indicate significant differences in photosynthesis and stomatal conductance in the *Trichoderma*-treated versus control plants using the Student’s *t*-test at a ^∗^*P* ≤ 0.05, ^∗∗^*P* ≤ 0.01 and ^∗∗∗^*P* ≤ 0.001. Bars represent the standard error.

The physiological parameters (photosynthesis rate and stomatal conductance) were measured to determine if *Trichoderma* has potential to improve physiological conditions in addition to plant aboveground and belowground biomass. The application of *Trichoderma* significantly increased the photosynthesis rate and stomatal conductance in only 5 and 4 genotypes, respectively, out of 15 tested (Figures 10A,B). The remaining genotypes were non-responsive to the presence of *Trichoderma*, and none of the genotypes were negatively affected by the *Trichoderma* treatment. The photosynthesis rate and stomatal conductance were increased by up to 57 and 114%, respectively, in responsive *Trichoderma* treated plants when compared with their respective non-treated control plants.

The nutrient contents (N contents and C:N ratio) were analyzed to determine if there are any correlations between changes in nutrient contents and disease reduction by *Trichoderma*. The total N content and C:N ratio in the biomass of the majority of the tomato genotypes were not significantly affected by *Trichoderma* treatment (except in LA1223 and LA1268 where N content increased, and in LA2951 where N content decreased and C:N ratio increased) (Supplementary Figure 2). The “domestication gradient” did have significant effects on the total N content and C:N ratio (Supplementary Table 1), however, the “*Trichoderma* treatment” and interaction between these two main factors was not significant. Similarly, breeding categories, *Trichoderma* treatment, and the interaction between these two main factors were not significant (Supplementary Table 2).

A correlation analysis between changes in nutrient contents (N contents and C:N ratio), *B. cinerea* and *P. infestans* disease reduction by *Trichoderma* indicated that there was no significant relationship between these factors (*P* > 0.05; Supplementary Figure 3).

### Correlation Between *Trichoderma*-Induced Total Phenolic and Flavonoid Contents and Systemic Resistance

Total phenolic and flavonoid content was determined in all sets of tomato genotypes to quantify the effect of domestication and breeding categories, and test if there is any relationship between the levels of the compounds and disease severity in order to ascertain if it is likely to be a mechanism underlying ISR. We found that “domestication gradient,” “*Trichoderma* treatment” and the interaction between these two main factors had significant effects on TFCs (Supplementary Table 1). However, only “domestication gradient” had a significant effect on TPC. There was no significant effect of “breeding category” on total phenolic or flavonoid content (Supplementary Table 2).

**FIGURE 11:**
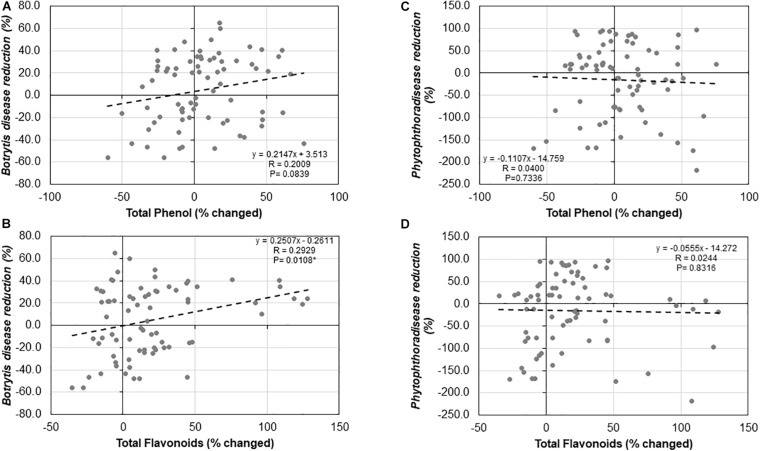
Correlation analysis between **(A)** *Botrytis* disease reduction (%) and changes in shoot total phenolic contents (%), **(B)** *Botrytis* disease reduction (%) and changes in total flavonoid contents (%), **(C)** *Phytophthora* disease reduction (%) and changed in total phenolic contents (%), and **(D)** *Phytophthora* disease reduction (%) and changed in total flavonoid contents (%) by *Trichoderma* treatment in comparison with the control treatment in 25 diverse tomato genotypes.

We observed no significant relationship between *Trichoderma*-induced TPC and ISR to *B. cinerea* (*R* = 0.2009, *P* = 0.0839; Figure 11A) or *P. infestans* (*R* = −0.0400, *P* = 0.7336; Figure 11C). Similarly, there was no significant correlation between the *Trichoderma*-induced TFC and ISR to *P. infestans* (*R* = −0.0244, *P* = 0.8316; Figure 11D). In contrast, there was a significant positive correlation between *Trichoderma*-induced TFC and ISR to *B. cinerea* (*R* = 0.2929, *P* = 0.0108; Figure 11B).

## Discussion

Identifying novel sources of disease resistance is critical to maintaining crop productivity while protecting environmental health. Consequently, interest in the use of beneficial microorganisms as an alternative to chemical pesticides has increased in the last decade. There have been many efforts to identify individual microbial taxa with disease suppressive activity, determine their mechanism of action and develop commercial products. Several of these biopesticides are now available in the marketplace, the majority of which contain either a single *Trichoderma* isolate or a mixture of *Trichoderma* species (Lopez-Bucio et al., 2015). However, the application of these microbial biopesticides are not widely accepted due to high variability in the efficacy of these products in the field (Lugtenberg and Leveau, 2007). Results of this study confirm earlier reports by Tucci et al. (2011) and Banani et al. (2014) in tomato and grapes respectively, that one reason for this variability could be differences in responsiveness among plant genotypes or varieties. Moreover, results of this study provide the first evidence that tomato domestication and breeding practices are important factors in mediating increases in plant growth and ISR to pathogens with distinct lifestyles (*Botrytis* and *Phytophthora*) in response to *Trichoderma.* These findings have important implications for the success of the emerging biopesticide industry. They also provide evidence that it might someday be possible to integrate selection for this beneficial plant-microbial relationship into breeding programs to reduce reliance on chemical pesticides and increase the productivity of tomato crops. Understanding why tomato genotypes appear to have become less responsiveness to beneficial soil microbes such as *Trichoderma*, is critical to realizing the benefits that can be conferred by these organisms in modern farming systems.

Previous studies have noted differences in early plant growth among maize (Harman et al., 2004b), tomato (Tucci et al., 2011) and chickpea (Bazghaleh et al., 2018) genotypes in response to *Trichoderma*, though ours is the first to demonstrate that plants have become less responsive to the plant growth benefits conferred by *Trichoderma* over domestication. Over the course of domestication, tomato plants have been selected for higher biomass and yield (Bai and Lindhout, 2007; Bergougnoux, 2014; Giovannoni, 2018). Consequently, it is not surprising that like other studies (Zsogon et al., 2018; Carrillo et al., 2019), we observed greater early basal plant growth parameters in modern cultivars relative to wild tomato germplasm. In some previous studies, differential responses among genotypes due to *Trichoderma* on early plant growth were negative in some cases (Harman et al., 2004b; Tucci et al., 2011). That was not the case in our study, where most genotypes had at least some increase in early plant growth in response to the *Trichoderma* inoculant. This difference between earlier studies and ours could have been due to the concentration and method of the *Trichoderma* inoculum used in our trial. All plant-associated microbes confer some cost to plants in terms of carbon allocation (Sasse et al., 2018), and high concentrations of what are normally considered ‘beneficial microbes’ can overwhelm plants and negatively affect growth and development (Suckstorff and Berg, 2003; Hoagland et al., 2012). Studies investigating regulation of mutualistic relationships with microbes such as mycorrhiza and rhizobia have demonstrated that some plants are able to control the amount of colonization, and even terminate these relationships when the partnership is no longer profitable and therefore negative (Werner et al., 2018). Thus, it is possible that some tomato plants have the potential to regulate colonization by *Trichoderma*, which could be responsible for the differential responses among genotypes observed.

Another potential reason for differences in the stimulatory effects of *Trichoderma* on early plant growth among genotypes involves nutrient acquisition. Previous studies have demonstrated that *Trichoderma* can enhance plant growth by solubilizing nutrients in soil, or directly enhancing the uptake of nitrogen and other nutrients by increasing root biomass (Harman et al., 2004a; Lopez-Bucio et al., 2015). In some crops, *Trichoderma* inoculation has reduced nitrogen fertilizer requirements by 40–50% with no associated reduction in crop yield (Harman et al., 2004a; Fiorentino et al., 2018). In natural ecosystems, plants do not receive fertilizers and therefore microbial symbionts play a critical role in helping them obtain nutrients (Porter and Sachs, 2020). Consequently, it is possible that the reason we observed a diminished growth response to the presence of *Trichoderma* over the course of domestication is that plants are no longer as reliant on these symbionts to obtain nutrients. This could also be the case for differences between tomato genotypes bred under organic and conventional conditions. Unlike in conventional production systems, where plants receive abundant supplies of inorganic fertilizers that are all readily available for plant uptake, plants in organic systems rely on organic fertility sources such as animal and plant residues that must mineralize before nutrients are available to plants (Hoagland et al., 2008; Rudisill et al., 2015; Reeve et al., 2016). Therefore, conducting breeding programs under organic conditions may favor selection for genotypes that are better able to support microbial symbionts like *Trichoderma* to help obtain nutrients. We did not observe differences in the total nitrogen content of leaves in response to *Trichoderma* in this study, though the plants were still young so it is possible that the nutrient benefits conferred by this microbe were not yet apparent. Future trials that allow tomato plants to grow to maturity should be conducted to test this hypothesis.

Plant hormones could also play a role in the differential responses observed in this study. *Trichoderma* strains are known to produce plant growth-regulators, such as auxin-like secondary metabolites: indole-3-acetic acid (IAA) and indole-3-acetaldehyde (IAAld) that stimulate root growth (Contreras-Cornejo et al., 2009; Chowdappa et al., 2013). Altering the balance between important hormones such as auxin, gibberellic acid and cytokinin can influence plant growth and development processes (Guzman-Guzman et al., 2019). We did not measure these hormones in our study, however, we speculate that there could be variability in the production or stimulation of these hormones by *Trichoderma* across tomato genotypes based on the results we obtained. For example, photosynthetic rate, stomatal conductance, and chlorophyll content as evaluated through leaf greenness were increased by *Trichoderma* in some but not all genotypes. Fungal pathogens and symbionts are theorized to produce hormonal compounds or stimulate their production in plants to favor colonization and nutrient acquisition, as well as to act as signals for their developmental or physiological processes (Chanclud and Morel, 2016). Consequently, if modern tomato genotypes no longer depend on fungal symbionts to obtain nutrients or promote other physiological processes, then it is possible they would evolve mechanisms to prevent colonization since they represent a cost to the plant. Alternatively, since some pathogens and symbionts tend to use overlapping colonization pathways (De Souza et al., 2016; Ramirez-Valdespino et al., 2019), selecting for resistance to biotrophic pathogens could have caused some genotypes to develop mechanisms to prevent colonization by fungi, regardless of their potential benefits.

Disease resistance has long been a critical target in varietal improvement efforts and tomato is no exception (Hoagland, 2015). Wild relatives and landraces of tomato are generally the best sources of resistance to biotic and abiotic stresses (Bergougnoux, 2014), and plant breeders often turn to these sources to enhance these traits in modern germplasm. Consequently, it was not surprising that the wild relatives of tomato evaluated in this trial were slightly more resistant to *B. cinerea* compared to other categories (*S*. *pimpinellifolium*, *S. cerasiforme* landrace/heirloom and modern cultivars). Basal resistance to *B. cinerea* has previously been reported in a number of wild relatives including accessions of *S. chilense* (Ten Have et al., 2007), *S. pennellii* (Smith et al., 2014), *S. habrochaites* (Finkers et al., 2007, 2008; Ten Have et al., 2007; Tucci et al., 2011), *S. neorickii* (Ten Have et al., 2007; Finkers et al., 2008), and *S. lycopersicoides* (Davis et al., 2009; Smith et al., 2014). However, in a recent study, Soltis et al. (2019) determined that resistance to a wide variety of *B. cinerea* isolates was largely independent of the influence of domestication. In the present study, we too observed no difference in basal resistance in *S. pimpinellifolium* compared to *S. lycopersicum* (landraces and modern cultivars). In addition, wild relatives and landraces were more susceptible to *P. infestans* than modern tomato genotypes. These results contrast with the general ‘domestication theory,’ which presumes that domesticated cultivars should be highly sensitive while also exhibiting reduced genetic diversity (Yamasaki et al., 2005; Gaillard et al., 2018; Ferrero et al., 2020). In the case of *P. infestans*, greater resistance in modern germplasm is likely due to targeted breeding efforts that have introgressed specific resistance genes (Ph-1, Ph-2, Ph-3, and Ph-5) (Foolad et al., 2008; Gardner and Panthee, 2010; Panthee and Gardner, 2010). In contrast, resistance to necrotrophic pathogens such as *Botrytis*, has been hampered by difficulties associated with introgressing resistance into elite germplasm without associated linkage drag (Smith et al., 2014). Unfortunately, we did not observe any influence of breeding practices on basal resistance against *Botrytis*, thus alternative approaches to help manage necrotrophic pathogens like this are needed to enhance the productivity of tomato crops. In contrast, some of the wild relatives evaluated in this trial, such as *S. pennellii* (LA0716) and *S. pimpinellifolium* (PI224710) were resistant to *P. infestans* indicating that they could hold value in future efforts to integrate further resistance into elite material. Other lines within *S. pennellii* and *S. pimpinellifolium* were susceptible *P. infestans* and thus would not have value for this trait. Interestingly, *S. pennellii* (LA0716) was resistant to both *B. cinerea* and *P. infestans*, indicating that this genotype could be particularly valuable for introgressing broad-spectrum resistance to both necrotrophic and hemibiotrophic pathogens.

Like previous studies (Martinelli et al., 1993; Chowdappa et al., 2013; Martinez-Medina et al., 2013), application of *Trichoderma* reduced the progression of disease caused by both *B. cinerea* and *P. infestans.* Several mechanisms for disease suppression have been observed in response to *Trichoderma* including mycoparasitism, antibiosis, competitive growth and ISR (Elad, 2000; Harman et al., 2004a). In the present study, the site of the *Trichoderma* treatment was physically separated from the site of pathogen inoculation (leaves). Therefore, we expect that reductions in disease severity were likely attributed to systemic resistance induced by *Trichoderma*. Furthermore, we screened for the presence of *Trichoderma* in tomato shoots using selective media (Elad and Chet, 1983), and were unable to detect its presence in any of the tomato genotypes (data not shown).

Many studies have demonstrated that plant genotype can affect the expression of induced resistance mediated by biological (Martinelli et al., 1993; Korolev et al., 2008; Banani et al., 2014; Alkooranee et al., 2015; Hossain and Sultana, 2015; Prashar and Vandenberg, 2017; Samain et al., 2019; Adam, 2020) and chemical (Herman et al., 2007; Ishikawa et al., 2007; Sharma et al., 2010, 2012; Walters et al., 2011; Cordova-Campos et al., 2012; Banani et al., 2014; Pawlowski et al., 2016) inducers in a wide variety of crops. Therefore, it is not surprising that like Tucci et al. (2011), we observed genetic differences in ISR by *Trichoderma* against *Botrytis*. However, we are the first to report that there were also differences among tomato genotypes in ISR to *P. infestans*, which has a very different lifestyle than *Botrytis*, and to document strong negative responses to *Trichoderma*. Tucci et al. (2011) also observed either a lack of an effect, or that *Trichoderma* could be detrimental against *Botrytis* in some genotypes, but that study only tested five genotypes (one wild and four modern). In our studies using a much wider set of tomato germplasm, *Trichoderma* inoculation reduced *B. cinerea* severity in 52% of the tomato genotypes, 8% were non-responsive, and in 40%, the fungus increased the severity of the pathogen. Interestingly, the majority of the negative responses, or lack of a response, were in the modern genotypes. Similar results were obtained with respect to late blight disease, where 44% of the tomato genotypes responded positively to *Trichoderma*; 32% responded negatively, and 24% were non-responsive to *Trichoderma*. Among the 25 tomato genotypes evaluated in this trial, only *S. pennellii* (LA1926), M-82 and Primavera were positively responsive to *Trichoderma* against both *B. cinerea* and *P. infestans*, whereas the hybrids and inbreds had negative responses to both pathogens. These results have critical implications for the biopesticide industry, by demonstrating that the effects of these microbes can positively, or negatively, affect resistance to diseases depending on the genotype planted. Elucidating the mechanisms regulating these processes is critical to overcoming this challenge and ensuring the continued growth and long-term success of the biopesticide industry.

One of the most significant results of our study, is the striking loss of responsiveness to *Trichoderma* mediated ISR over the course of tomato domestication. For example, the wild relatives, *S. cerasiforme*, landraces and heirloom tomatoes were more responsive to *Trichoderma* than modern genotypes against *B. cinerea*. Others have found that ISR mediated by benzothiadiazole (BTH, a chemical inducer) against two bacterial pathogens (*Pseudomonas syringae* pv. *syringae* and *Enterobacter* sp.) in *Phaseolus vulgaris* (common bean) was influenced by domestication (Cordova-Campos et al., 2012). Specifically, in that trial, the wild *Phaseolus* accession and a landrace were responsive, but the modern cultivars were not. Results of that study and ours provide evidence that new high-yielding modern cultivars have lost a considerable part of the induced broad-spectrum resistance that characterizes wild relatives and landraces. As we discussed above with respect to the gradual loss in the ability of tomato plants to reap the benefits of early plant growth induced by *Trichoderma*, this could be due to the fact that most modern cultivars have been developed in conventional agricultural systems. In these systems, plants receive inorganic fertilizers and, in some cases, pesticides are applied to control minor diseases, which could have caused beneficial plant-microbial relationships to be lost over time (Hoagland, 2015). The mechanisms responsible for this loss could be related to changes in plant root exudates. It is estimated that up to 40% of the carbon photosynthetically fixed by plants is exuded as rhizodeposits (e.g., exudates, border cells, mucilage), which play important roles in chemotropism, colonization and growth of microbes in the rhizosphere (Bais et al., 2006). Iannucci et al. (2017) reported that root exudate profiles have changed over domestication in wheat and Carrillo et al. (2019) observed greater increases in soil microbial biomass in wild relative to modern tomato genotypes. Thus, it is possible that the differences we observed in this trial were caused by changes in plant root exudate profiles. Another possibility is that the tomato genotypes differ in the perception of effectors molecules used to identify and permit colonization of the fungus. Ramirez-Valdespino et al. (2019) recently reported that these effector molecules are important in mediating *Trichoderma* relationships with their plant host. Finally, differential responses among the tomato genotypes in response to *Trichoderma* mediated ISR, could be due to the production of secondary metabolites by the fungus, which could be detrimental to some genotypes and interfere with hormonal crosstalk that is important for controlling beneficial and pathogenic relationships.

In the present study, we observed a trade-off between *Trichoderma*-mediated ISR to pathogens with two very distinct lifestyles (i.e., necrotrophic vs. hemi-biotrophic). It is generally assumed that immunity to necrotrophic pathogens and chewing herbivores is mediated by the jasmonic acid (JA) pathway, while resistance to (hemi-)biotrophic pathogens and phloem-feeding insects is mediated by salicylic acid (SA) pathways (Glazebrook, 2005; Spoel et al., 2007; Rahman et al., 2012). Many studies have demonstrated that there can be antagonistic crosstalk between the SA- and JA-defense pathways (Robert-Seilaniantz et al., 2011; Yang et al., 2019), meaning that activation of the JA pathway can result in suppression of SA-induced responses, and vice-versa. *Trichoderma* spp.-mediated ISR is generally associated with JA and ET biosynthesis and signaling pathways (Shoresh et al., 2005, 2010), however, in some cases, *Trichoderma* spp. have been demonstrated to induce SA and ET pathways (Hermosa et al., 2012; Harel et al., 2014; Leonetti et al., 2017). Consequently, variability among tomato genotypes in responsiveness to IR by *Trichoderma* against the two distinct pathogens evaluated in this trial could be due to differential responses in the activation of these defense-related genes and their crosstalk (Tucci et al., 2011; Martinez-Medina et al., 2013). For example, we observed strong negative correlations between basal resistance and induced resistance for both *B. cinerea* and *P. infestans*. This suggests that induction of systemic resistance by *Trichoderma* was greater in susceptible tomato genotypes than in resistant. Similar negative relationship between levels of basal and induced resistance has been observed in bean (Cordova-Campos et al., 2012), tomato (Tucci et al., 2011), *Arabidopsis* (Ton et al., 2001; Hossain and Sultana, 2015), cucumber (Hijwegen and Verhaar, 1995; Liu et al., 1995) and tobacco (Perez et al., 2003). In contrast, induced resistance was greater in resistant than susceptible grapevine cultivars (Banani et al., 2014), but no relationship between levels of basal and induced resistance were observed in tomato (Sharma et al., 2010), or spring barley (Walters et al., 2011). Determining whether variability in the biosynthesis and signal transduction of defense genes that underlie plant genetic control of the interactions was beyond the scope of this study, but should be explored in future studies.

Finally, we suspected that changes in N concentrations, total phenolic and flavonoid compounds induced by *Trichoderma* could have been a factor in mediating plant defense responses against *B. cinerea* and *P. infestans.* However, we observed no evidence that changes in total N or total phenolic concentration could explain *Trichoderma*-mediated systemic resistance against *P. infestans* or *B. cinerea*. In contrast, we did observe a positive relationship between *Trichoderma*-mediated systemic resistance against *B. cinerea* and *Trichoderma*-enhanced TFC. Similarly, Mathys et al. (2012) found induction of reactive oxygen species (ROS) and increased production of antioxidants (anthocyanins and flavonoids) during *Trichoderma*-mediated ISR in *Arabidopsis thaliana*. These authors suggested that this could explain why they observed reductions in plant cell necrosis, a process which is considered favorable for necrotrophic pathogens such as *B. cinerea* (Mathys et al., 2012). Consequently, it is possible that the differential response among tomato genotypes in *Trichoderma* mediated suppression of *Botrytis* is due to their potential to upregulate production of flavonoids.

## Conclusion

Our results demonstrate that both basal and induced plant growth and systemic resistance to foliar pathogens have been affected by domestication and breeding practices. This could help explain why the efficacy of biopesticides is often so variable in field trials. In addition, these results provide evidence for the potential to enhance the efficacy of these products, reduce reliance on chemical pesticides and improve the productivity of tomato crops by selecting varieties that are responsive to the presence of these microbes. This will require further efforts to decipher the mechanisms behind these differences in the induction of resistance among genotypes, and identify the molecular markers that can be used to efficiently select for this trait in breeding programs.

## Data Availability Statement

The original contributions presented in the study are included in the article/Supplementary Material, further inquiries can be directed to the corresponding author.

## Author Contributions

AJ participated in experimental planning, conducted all experiments, data collection and analysis, and took the lead in writing the manuscript. TM, JM, DE, and LH participated in experimental planning, data interpretation, and contributed to writing the manuscript. LH also obtained the funding to conduct the research and all experiments were conducted in their lab. All authors contributed to the article and approved the submitted version.

## Conflict of Interest

The authors declare that the research was conducted in the absence of any commercial or financial relationships that could be construed as a potential conflict of interest.

## Footnotes

1 http://www.fao.org/faostat/

2 https://eorganic.info/tomi

3 https://imagej.nih.gov/ij/
