# Supplementary material for: Tomato Domestication Attenuated Responsiveness to a Beneficial Soil Microbe for Plant Growth Promotion and Induction of Systemic Resistance to Foliar Pathogens
Source: Front Microbiol. 2020 Dec 18;11:604566. doi: 10.3389/fmicb.2020.604566 (PMC7775394; doi:10.3389/fmicb.2020.604566)
Supplement: Supplementary file 1 [file Data_Sheet_1.docx]

***Supplementary Material***

**Supplementary Table S1** Analysis of variance (ANOVA) to evaluate interactions between tomato domestication and *Trichoderma* treatment on plant growth, disease reduction, biochemical compounds and nutrient contents. Asterisks indicate significant at *P*≤ 0.05.

| Source | Root weight (g)^a^ | Plant height (cm) | Canopy dry weight (g) | Leaves number | Leaf greenness | *Botrytis* disease (mm^2^) | *Phytophthora* disease (mm^2^) | Total Phenol (mg GAE/100g leaves) | Total Flavonoid (mg QE/100 g leaves) | N (mg/g dry leaf) | C/N ratio |
| --- | --- | --- | --- | --- | --- | --- | --- | --- | --- | --- | --- |
| ANOVA |  |  |  |  |  |  |  |  |  |  |  |
| Model | <0.0001* | <0.0001* | <0.0001* | <0.0001* | <0.0001* | <0.0001* | <0.0001* | <0.0001* | <0.0001* | <0.0001* | <0.0001* |
| R^2^ | 0.5674 | 0.5295 | 0.5362 | 0.2925 | 0.2946 | 0.3281 | 0.3965 | 0.2559 | 0.6028 | 0.4796 | 0.4032 |
| *P*-value |  |  |  |  |  |  |  |  |  |  |  |
| Domestication (D) | <0.0001* | <0.0001* | <0.0001* | <0.0001* | <0.0001* | <0.0001* | <0.0001* | <0.0001* | <0.0001* | <0.0001* | <0.0001* |
| *Trichoderma* (T) | <0.0001* | <0.0001* | <0.0001* | <0.0001* | <0.0001* | 0.0068* | 0.0501 | 0.4099 | <0.0001* | 0.8952 | 0.8834 |
| D×T | 0.0009* | 0.0150* | 0.3925 | 0.2950 | 0.9651 | 0.0445* | 0.0101* | 0.3275 | 0.0004* | 0.9348 | 0.9801 |

**Supplementary Table S2** Analysis of variance (ANOVA) to quantify interactions between tomato breeding conditions and *Trichoderma* treatment on plant growth, disease reduction, biochemical compounds and nutrient contents. Asterisks indicate significant at *P*≤ 0.05.

| Source | Root weight (g)^a^ | Plant height (cm) | Canopy dry weight (g) | Leaves number | Leaf greenness | *Botrytis* disease (mm^2^) | *Phytophthora* disease (mm^2^) | Total Phenol (mg GAE/100g leaves) | Total Flavonoid (mg QE/100 g leaves) | N (mg/g dry leaf) | C/N ratio |
| --- | --- | --- | --- | --- | --- | --- | --- | --- | --- | --- | --- |
| ANOVA |  |  |  |  |  |  |  |  |  |  |  |
| Model | <0.0001^*^ | 0.0850 | 0.0009^*^ | 0.1019 | 0.0001^*^ | 0.4980 | 0.0185^*^ | 0.0523 | 0.9624 | 0.4405 | 0.0881 |
| R^2^ | 0.2322 | 0.0753 | 0.1781 | 0.0708 | 0.2197 | 0.0373 | 0.1480 | 0.1161 | 0.0046 | 0.0314 | 0.0744 |
| *P*-value |  |  |  |  |  |  |  |  |  |  |  |
| Breeding (B) | 0.1014 | 0.7501 | 0.7422 | 0.0765 | 0.0008^*^ | 0.2203 | 0.0666 | 0.2539 | 0.7194 | 0.2116 | 0.0316^*^ |
| *Trichoderma* (T) | <0.0001^*^ | 0.0160^*^ | <0.0001^*^ | 0.1211 | 0.0017^*^ | 0.3590 | 0.0366^*^ | 0.2855 | 0.8767 | 0.6121 | 0.5467 |
| B×T | 0.9729 | 0.2943 | 0.6195 | 0.4786 | 0.4460 | 0.8328 | 0.0708 | 0.0164^*^ | 0.7301 | 0.3291 | 0.1900 |

**Supplementary Figure S1** Effect of *Trichoderma* treatment on (A) leaf greenness (%) in a diverse set of tomato genotypes. Graph (B) represents the percentage in leaf greenness changed by *Trichoderma* treatment in comparison with the control treatment. Asterisks indicate significant differences in leaf greenness in the *Trichoderma*-treated versus control plants using the Student’s t-test at a *P*≤ 0.05 (*), *P*≤ 0.01 (**) and *P*≤ 0.001 (***). Bars represent the standard error.

**Supplementary Figure S2** Effects of *Trichoderma* treatment on (A) N content and (B) C:N ratio in a set of diverse tomato genotypes. Asterisks indicate significant differences in nutrient in the *Trichoderma*-treated versus control plants using the Student’s t-test at a *P*≤ 0.05 (*), *P*≤ 0.01 (**) and *P*≤ 0.001 (***). Bars represent the standard error.

**Supplementary Figure S3** Correlation analysis between (A) *Botrytis* disease reduction (%) and changed in shoot N contents (%), (B) *Botrytis* disease reduction (%) and changed in shoot C:N ratio (%), (C) *Phytophthora* disease reduction (%) and changed in shoot N contents (%), and (D) *Phytophthora* disease reduction (%) and changed in shoot C:N ratio (%) by *Trichoderma* treatment in comparison with the control treatment in 25 diverse tomato genotypes.

**Supplementary Table S3** Effect of *Trichoderma* treatment on root biomass and plant height in a diverse set of tomato genotypes. *P-Value* within each genotype (row) indicates significant differences in root biomass and plant height in the *Trichoderma*-treated versus control plants at *P* ≤ 0.05 using the Student’s t-test.

|  | Root biomass (g) | | | | Plant height (cm) | | | |
| --- | --- | --- | --- | --- | --- | --- | --- | --- |
| Genotype | Control | *Trichoderma* | % increased | *P-Value* | Control | Trichoderma | % increased | *P-Value* |
| *S.lycoides* LA2951 | 0.5 ± 0.19 | 1.6 ± 0.19 | 250.0 | 0.0059 | 32.3 ± 2.56 | 36.8 ± 2.14 | 14.0 | 0.2260 |
| *S.pennellii* LA0716 | 0.5 ± 0.14 | 2.8 ± 0.43 | 423.8 | 0.0028 | 20.5 ± 1.19 | 28.3 ± 1.70 | 37.8 | 0.0097 |
| *S.pennellii* LA1926 | 0.9 ± 0.53 | 4.7 ± 0.83 | 434.3 | 0.0084 | 12.3 ± 1.93 | 23.5 ± 3.07 | 91.8 | 0.0210 |
| *S.Chilense* LA1932 | 1.5 ± 0.51 | 9.6 ± 1.12 | 526.2 | 0.0006 | 28.0 ± 2.12 | 38.8 ± 0.75 | 38.4 | 0.0031 |
| *S.Habro* LA1223 | 5.8 ± 0.29 | 9.5 ± 0.88 | 62.7 | 0.0076 | 28.8 ± 2.87 | 38.0 ± 0.82 | 32.2 | 0.0211 |
| *S.pimpi* LA1589 | 3.8 ± 0.98 | 13.8 ± 1.16 | 266.2 | 0.0006 | 38.5 ± 3.93 | 63.3 ± 3.97 | 64.3 | 0.0044 |
| *S.pimpi* PI224710 | 6.7 ± 1.12 | 11.6 ± 0.74 | 72.4 | 0.0113 | 30.8 ± 0.95 | 44.0 ± 2.94 | 43.1 | 0.0052 |
| *S.lyco.*Wild LA1231 | 7.7 ± 0.61 | 13.1 ± 0.63 | 70.0 | 0.0008 | 38.0 ± 0.82 | 46.0 ± 0.71 | 21.1 | 0.0003 |
| *S.lyco.*Wild LA1268 | 8.8 ± 0.30 | 18.1 ± 1.26 | 105.4 | 0.0004 | 37.0 ± 1.78 | 46.5 ± 1.32 | 25.7 | 0.0052 |
| *S.lyco.*Wild LA2845 | 5.9 ± 0.54 | 9.0 ± 0.64 | 51.9 | 0.0104 | 37.8 ± 1.31 | 42.5 ± 1.89 | 12.6 | 0.0850 |
| *S.lyco.*Matt Wild Cherry | 4.6 ± 0.41 | 12.8 ± 0.71 | 180.8 | <0.0001 | 30.0 ± 1.08 | 37.5 ± 1.32 | 25.0 | 0.0046 |
| *S.lyco.*Land LA0134C | 9.2 ± 0.60 | 21.1 ± 1.58 | 130.6 | 0.0004 | 46.3 ± 2.29 | 54.8 ± 2.29 | 18.4 | 0.0391 |
| *S.lyco.*HL Brandywine | 5.3 ± 0.91 | 7.6 ± 0.62 | 44.8 | 0.076 | 40.5 ± 1.04 | 45.8 ± 1.03 | 13.0 | 0.0116 |
| *S.lyco.*HL Corbarino | 12.4 ± 1.18 | 22.4 ± 1.94 | 80.6 | 0.0046 | 61.0 ± 1.29 | 69.3 ± 1.93 | 13.5 | 0.0120 |
| *S.lyco.*EM Crimson Sprinter | 4.7 ± 0. 50 | 5.2 ± 0.80 | 10.1 | 0.6333 | 52.0 ± 1.68 | 53.8 ± 2.29 | 3.4 | 0.5603 |
| *S.lyco.*EM Wisconsin55 | 7.1 ± 1.25 | 7.8 ± 0.87 | 10.2 | 0.6513 | 39.8 ± 1.18 | 46.8 ± 1.31 | 17.6 | 0.0411 |
| *S.lyco*.EM M82 | 6.5 ± 0.45 | 9.6 ± 0.89 | 48.1 | 0.046 | 30.0 ± 0.82 | 31.3 ± 1.31 | 4.2 | 0.4502 |
| *S.lyco.*F1 Iron Lady | 7.7 ± 0.62 | 14.1 ± 0.80 | 83.1 | 0.0008 | 39.3 ± 2.50 | 54.0 ± 2.12 | 37.6 | 0.0041 |
| *S.lyco*/inb NC2CELBR | 9.2 ± 0.77 | 12.3 ± 1.18 | 33.9 | 0.0701 | 41.0 ± 1.08 | 46.5 ± 0.96 | 13.4 | 0.0560 |
| *S.lyco.*Org Primavera | 8.4 ± 0.94 | 10.2 ± 0.76 | 22.5 | 0.1727 | 52.5 ± 2.06 | 53.5 ± 2.10 | 1.9 | 0.7457 |
| *S.lyco.*Org Clou | 4.1 ± 0.31 | 5.5 ± 0.83 | 32.7 | 0.1803 | 50.8 ± 1.89 | 53.3 ± 1.97 | 4.9 | 0.3953 |
| *S.lyco.*Org T-1807 | 6.5 ± 0.79 | 7.9 ± 1.32 | 21.2 | 0.4056 | 34.3 ± 1.31 | 37.8 ± 1.93 | 10.2 | 0.1848 |
| *S.lyco.*Org T-1809 | 6.7 ± 0.43 | 12.0 ± 1.13 | 79.4 | 0.0047 | 36.0 ± 0.58 | 40.8 ± 1.11 | 13.2 | 0.0090 |
| *S.lyco.*Org T-1815 | 5.9 ± 0.88 | 10.6 ± 1.44 | 78.9 | 0.0328 | 38.0 ± 1.00 | 38.5 ± 1.19 | 1.3 | 0.7586 |
| *S.lyco*.Org T-1820 | 4.6 ± 0.79 | 6.9 ± 0.81 | 48.1 | 0.0976 | 38.5 ± 1.55 | 40.8 ± 1.44 | 5.8 | 0.3286 |

**Supplementary Table S4** Effect of *Trichoderma* treatment on *Botrytis* and *Phytophthora* disease severity in a diverse set of tomato genotypes. *P-Value* within each genotype (row) indicates significant differences in disease severity in the *Trichoderma*-treated versus control plants at *P* ≤ 0.05 using the Student’s t-test.

|  | *Botrytis* disease severity (mm^2^) | | | | Phytophthora disease severity (mm^2^) | | | |
| --- | --- | --- | --- | --- | --- | --- | --- | --- |
| Genotype | Control | *Trichoderma* | CE (%) | *P-Value* | Control | Trichoderma | CE (%) | *P-Value* |
| *S.lycoides* LA2951 | 10.8 ± 0.68 | 15.5 ± 0.83 | -42.5 | 0.0126 | 148.1 ± 9.65 | 9.3 ± 1.45 | 93.7 | 0.0001 |
| *S.pennellii* LA0716 | 12.8 ± 1.00 | 8.4 ± 0.81 | 34.5 | 0.0266 | 2.9 ± 0.54 | 7.4 ± 0.43 | -153.1 | 0.0031 |
| *S.pennellii* LA1926 | 15.9 ± 1.12 | 11.9 ± 0.53 | 25.1 | 0.0328 | 86.1 ± 7.74 | 18.2 ± 3.49 | 78.9 | 0.0013 |
| *S.Chilense* LA1932 | 8.1 ± 0.86 | 9.4 ± 0.57 | -16.9 | 0.2553 | 71.1 ± 5.45 | 47.5 ± 4.41 | 33.2 | 0.0282 |
| *S.Habro* LA1223 | 20.7 ± 1.10 | 9.1 ± 1.35 | 56.2 | 0.0026 | 86.1 ± 3.00 | 96.2 ± 9.53 | -11.7 | 0.3706 |
| *S.pimpi* LA1589 | 17.5 ± 0.53 | 10.7 ± 0.33 | 38.7 | 0.0004 | 117.8 ± 6.12 | 115.6 ± 8.42 | 1.9 | 0.8434 |
| *S.pimpi* PI224710 | 16.7 ± 1.00 | 13.0 ± 0.50 | 22.1 | 0.0301 | 9.7 ± 2.04 | 13.5 ± 2.78 | -39.7 | 0.3287 |
| *S.lyco.*Wild LA1231 | 23.3 ± 1.16 | 13.4 ± 0.86 | 42.5 | 0.0024 | 45.2 ± 3.72 | 68.0 ± 7.08 | -50.6 | 0.0461 |
| *S.lyco.*Wild LA1268 | 13.9 ± 0.67 | 17.0 ± 0.81 | -22.5 | 0.041 | 79.3 ± 12.97 | 13.0 ± 7.00 | 83.6 | 0.0108 |
| *S.lyco.*Wild LA2845 | 16.2 ± 0.61 | 10.0 ± 0.34 | 38.7 | 0.0008 | 35.8 ± 8.67 | 101.4 ± 6.54 | -183.1 | 0.0038 |
| *S.lyco.*Matt Wild Cherry | 19.4 ± 1.68 | 12.5 ± 1.24 | 35.6 | 0.0296 | 14.9 ± 5.23 | 23.3 ± 4.15 | -56.7 | 0.2746 |
| *S.lyco.*Land LA0134C | 9.8 ± 0.56 | 12.9 ± 0.70 | -32.1 | 0.0253 | 83.4 ± 6.06 | 63.5 ± 2.65 | 23.9 | 0.0394 |
| *S.lyco.*HL Brandywine | 20.7 ± 1.89 | 14.4 ± 0.77 | 30.6 | 0.036 | 55.8 ± 8.15 | 95.5 ± 6.50 | -71.0 | 0.019 |
| *S.lyco.*HL Corbarino | 10.8 ± 0.29 | 7.7 ± 0.18 | 28.9 | 0.0008 | 54.3 ± 7.26 | 86.8 ± 6.12 | -60.1 | 0.0265 |
| *S.lyco.*EM Crimson Sprinter | 28.3 ± 0.78 | 33.3 ± 1.55 | -17.6 | 0.0454 | 105.3 ± 9.51 | 27.8 ± 6.95 | 73.6 | 0.0028 |
| *S.lyco.*EM Wisconsin55 | 22.9 ± 1.00 | 21.7 ± 0.77 | 5.1 | 0.4074 | 32.8 ± 5.57 | 3.3 ± 0.67 | 89.8 | 0.0063 |
| *S.lyco*.EM M82 | 15.4 ± 0.95 | 12.0 ± 0.76 | 21.9 | 0.0501 | 10.8 ± 0.78 | 7.5 ± 0.76 | 30.4 | 0.0397 |
| *S.lyco.*F1 Iron Lady | 11.8 ± 0.28 | 14.6 ± 0.72 | -23.7 | 0.022 | 2.6 ± 0.29 | 4.5 ± 0.58 | -76.1 | 0.0399 |
| *S.lyco*/inb NC2CELBR | 10.7 ± 0.63 | 14.9 ± 1.27 | -38.6 | 0.0428 | 5.0 ± 0.50 | 11.8 ± 1.30 | -136.1 | 0.0081 |
| *S.lyco.*Org Primavera | 19.5 ± 0.75 | 15.0 ± 0.71 | 23.3 | 0.012 | 15.9 ± 0.63 | 10.9 ± 0.58 | 31.4 | 0.0042 |
| *S.lyco.*Org Clou | 13.7 ± 0.77 | 18.7 ± 1.03 | -36.7 | 0.0173 | 27.8 ± 2.17 | 23.7 ± 0.93 | 14.9 | 0.1534 |
| *S.lyco.*Org T-1807 | 16.6 ± 1.38 | 22.5 ± 0.77 | -35.8 | 0.0199 | 8.2 ± 0.40 | 5.5 ± 0.58 | 33.1 | 0.0179 |
| *S.lyco.*Org T-1809 | 27.5 ± 1.44 | 20.6 ± 1.07 | 25.0 | 0.0183 | 2.7 ± 0.67 | 4.2 ± 1.17 | -56.3 | 0.3268 |
| *S.lyco.*Org T-1815 | 20.9 ± 0.36 | 23.6 ± 0.41 | -12.9 | 0.0079 | 14.3 ± 1.67 | 7.3 ± 0.88 | 48.8 | 0.0206 |
| *S.lyco*.Org T-1820 | 18.0 ± 0.70 | 22.4 ± 0.74 | -24.5 | 0.0124 | 5.7 ± 1.20 | 11.8 ± 1.01 | -108.8 | 0.0172 |
